# Development and interpretation of a machine learning model for predicting body mass index in Chinese adolescents: a prospective cohort study

**DOI:** 10.3389/fpubh.2025.1657551

**Published:** 2025-11-20

**Authors:** Zikang Zhang, Wei Peng, Shaoming Sun, Fangwen Zhang, Yining Sun, Lei Huang

**Affiliations:** 1 Hefei Institutes of Physical Science, Chinese Academy of Sciences, Hefei, Anhui, China; 2 University of Science and Technology of China, Hefei, Anhui, China; 3 CAS Hefei Institute of Technology Innovation, Hefei, Anhui, China

**Keywords:** BMI, prediction model, machine learning, daily information, model interpretation, modifiable factors

## Abstract

**Purposes:**

This study aimed to develop a machine learning model to predict body mass index (BMI) in adolescents based on readily accessible daily information and to investigate the influence of modifiable factors on BMI changes through model interpretation techniques.

**Methods:**

This study is a one-year prospective cohort study. Baseline data were collected through anthropometric measurements and questionnaires, and BMI were reassessed after 1 year. Six machine learning models were developed to predict BMI. Nested cross-validation (CV) was used for hyperparameter tuning and performance estimation. Predictors were prescreened on the inner-training folds of the nested CV using univariable analyses. Model performance was evaluated using Root Mean Squared Error (RMSE), Mean Squared Error (MSE), Mean Absolute Error (MAE), and coefficient of determination (R^2^). SHapley Additive exPlanations (SHAP) was used for global and local interpretations of the models.

**Results:**

The mean BMI of the 1,827 students included in the final analysis increased from 21.18 ± 3.63 kg/m^2^ at baseline to 21.54 ± 3.59 kg/m^2^ after 1 year, with an average change of 0.36 ± 1.40 kg/m^2^. The CatBoost (CB) model demonstrated the best predictive performance. After calibration, it achieved an RMSE of 1.200 [95% confidence interval (CI): 1.101–1.303], MSE of 1.440 (95% CI: 1.211–1.697), MAE of 0.895 (95% CI: 0.818–0.981) and R^2^ of 0.902 (95% CI: 0.882–0.918). In the SHAP analysis, the top 5 modifiable features at the population level were: level of health literacy, recognize self-weight status correctly, sedentariness duration on weekends, participation in professional sports training, frequency of staying up late.

**Conclusion:**

This study developed a BMI prediction model for adolescents using readily accessible daily information. The model accurately predicts BMI values 1 year later and provides both population-level and individual-level interpretability. Compared to existing studies, it offers key advantages, including independence from complex clinical data, the ability to predict continuous BMI values, and strong model interpretability. Our findings provide a promising research tool for screening high-risk adolescents, informing public health prevention and intervention strategies, and supporting personalized clinical interventions.

## 1 Introduction

Body mass index (BMI) is a commonly used indicator for assessing the ratio of an individual’s weight to height, widely applied in public health to help identify early health risks (1). Abnormal BMI values are closely associated with the onset of various chronic diseases, such as cardiovascular diseases, diabetes, and certain types of cancer (2–5). According to the 2024 Global Burden of Disease Study, high BMI (≥25 kg/m^2^) is a major risk factor for non-communicable diseases, contributing to 5–42% of related deaths and 5–52% of disability-adjusted life years (6). By 2020, the prevalence of high BMI among Chinese children and adolescents aged 5–19 had reached 37% and was projected to rise to 72% by 2035 at an average annual growth rate of 2.0%, with more than 31.5 million expected to develop health problems related to non-communicable diseases (6). Given the alarming rise in abnormal BMI rates, Chinese children and adolescents represent a large and rapidly growing at-risk population, making BMI-related research in this group highly valuable for public health. Early BMI trajectory identification offers key opportunities for timely interventions to prevent weight-related health risks.

BMI in children and adolescents is influenced by a wide array of factors, including genetic, behavioral, psychological, dietary, familial, school-related, and sociodemographic factors, as supported by previous research (7–9). Given this multifactorial nature, accurately predicting BMI requires methods that can accommodate diverse and potentially nonlinear influences. Traditional statistical approaches are often constrained by strong parametric assumptions, limiting their ability to model such complex interactions. In contrast, machine learning techniques offer greater flexibility and are particularly adept at uncovering deeper connections between features in health-related data, making them well-suited for BMI prediction. In current research, machine learning-based BMI prediction models have been developed using a diverse array of predictive indicators, including medication data, biological markers, body images, smartphone motion sensor data, and lifestyle-related information (10–15). However, current models often depend on complex and hard-to-collect predictors, which limits their scalability and real-world applicability. Meanwhile, some approaches that incorporate daily information still primarily classify obesity status rather than predict continuous BMI values, reducing their ability to capture subtle changes.

Interpretability has become an increasingly important focus in machine learning prediction models, leading to their growing application across diverse clinical contexts (16). By enhancing model transparency and interpretability, they support understanding of prediction logic and assist clinical decision-making, thereby narrowing the gap between model development and practical application. Among available interpretation techniques, SHapley Additive exPlanations (SHAP) is theoretically grounded in cooperative game theory (17), providing consistent and locally accurate feature attributions, which makes it particularly suitable for individualized interpretation in health-related prediction tasks. SHAP has been successfully applied to the interpretation of clinical prediction models, including frailty, myelosuppression risk, and acute kidney injury in pediatric cardiac surgery patients (18–20). However, the application of model interpretability techniques in BMI research remains limited, hindering a deeper understanding of how various predictors influence BMI predictions and limiting the practical utility of these models in real-world scenarios.

The main contributions of this paper can be summarized as follows:

1. We developed a machine learning model to predict the BMI of Chinese adolescents using only readily accessible daily information. This approach overcomes the limitations of previous studies that relied heavily on clinical biomarkers or complex datasets. It offers an efficient and cost-effective solution for early identification of high-risk adolescents, with potential for use in community, school, and clinical settings.
2. We focused on predicting continuous BMI values rather than merely classifying weight status, enabling the detection of subtle changes in body weight. These minor fluctuations can indicate early-stage health risks, which are clinically important for timely interventions, dynamic health monitoring, and chronic disease prevention.
3. We utilized model interpretability techniques to uncover the impact of modifiable factors on BMI variations at both group and individual levels. This interpretability not only enhances policymakers’ decision-making efficiency in weight management and health promotion, but also provides clinicians with targeted, personalized intervention strategies, offering substantial practical value.

## 2 Literature review

Extensive empirical studies have clarified the key determinants of BMI. Silventoinen et al. examined the genetic and environmental contributions to BMI variation from infancy to early adulthood, revealing that genetic factors played a major role in BMI variation during adolescence, while environmental factors influenced childhood BMI (7). Zink et al. found significant longitudinal associations between screen time, physical activity, sleep duration, and BMI in U. S. youth (8). Sandri et al. conducted a study to explore the impact of sociodemographic, nutritional, and lifestyle factors on BMI in Spain, highlighting the role of poor dietary habits and sociodemographic characteristics in influencing obesity risk (9).

Recent research has applied various machine learning techniques to predict BMI, using a wide range of predictive factors. Park et al. identified specific brain regions’ functional connectivity as significant biomarkers for predicting BMI changes in adolescents, with high accuracy achieved through machine learning-based neuroimaging analysis (10). Yao et al. proposed a deep learning model that uses smartphone motion sensors to predict BMI, demonstrating that motion entropy-based filtering significantly improved the model’s prediction accuracy, particularly with jogging as the activity of choice (12). Kim et al. presented an approach for predicting BMI and various body part sizes using multi-view body images. Their method demonstrated high accuracy and highlighted the potential of leveraging large-scale open datasets for applications in health monitoring, fitness tracking, and apparel sizing (13). Arumäe et al. found that while the five personality domains could predict current BMI, 29 specific personality traits were able to predict both current and future BMI (21). Singh and Tawfik reported that early BMI data, along with demographic factors such as age and gender, serve as key predictors for forecasting BMI changes during adolescence (22).

Recent studies have increasingly incorporated interpretability techniques to enhance transparency in machine learning-based health predictions. Li et al. developed an individualized prediction model for myelosuppression risk in lung cancer patients using machine learning, employing SHAP to evaluate feature importance, with the analysis indicating white blood cell count, platelet count, neutrophil count, BMI, and age as the most influential predictors (18). Luo et al. trained machine learning models to predict cardiac surgery-associated acute kidney injury (CSA-AKI) in pediatric patients, utilizing SHAP to identify key predictors such as baseline serum creatinine level, perfusion time, and operation time (19). Yu et al. applied SHAP to interpret the LightGBM model for predicting frailty risk, emphasizing the importance of cognitive function, grip strength, sleep duration, and BMI as key predictors, and demonstrated SHAP’s effectiveness in revealing the model’s decision-making process (20).

## 3 Methods

### 3.1 Selection of participants

This study, conducted in September 2023 in Anhui, China, involved students aged 14–17 from nine pilot high schools. These pilot schools included both general senior high schools and vocational schools, and were located in central and non-central cities, ensuring the inclusion of students with diverse socioeconomic backgrounds. The inclusion criteria required participants to meet the following conditions: (1) no history of major illnesses; (2) no plans to transfer schools or relocate during the upcoming year; (3) the ability to participate in follow-up surveys for 1 year.

### 3.2 Data collection

At baseline, participants underwent anthropometric assessments, including body mass and height measurements with digital scales and wall-mounted stadiometers, conducted by trained researchers. BMI was calculated as weight in kilograms divided by height in meters squared $(\mathrm{BMI}=\frac{\text{weight}\;(\mathrm{kg})}{\text{heigh}t^{2}(m^{2})}).$ Basal metabolic rate (BMR) was estimated using the FAO/WHO/UNU adolescent (10–18 y) predictive equations: for male, BMR (kcal/day) = 16.6 × Weight (kg) + 77 × Height (m) + 572; for female, BMR (kcal/day) = 7.4 × Weight (kg) + 482 × Height (m) + 217 (23, 24). Overweight and obesity status was classified based on age- and sex-specific BMI reference standards established by the Working Group on Obesity in China for school-aged children and adolescents (25). In addition, students and their parents completed a structured electronic questionnaire through a publicly accessible online system available on both mobile phones and computers. Developed based on prior studies (7–9, 26, 27), the questionnaire covered a range of factors, including genetic predispositions, socio-demographic characteristics, daily habits, physical activity patterns, self-perception of body status, and health literacy. The system required participants to complete all items before submission, ensuring no missing data at the individual question level. After 1 year, a follow-up anthropometric assessment was conducted to evaluate BMI changes in the cohort, with assessors blinded to baseline measurements.

### 3.3 Statistical analysis

This study conducted a power analysis based on a medium effect size (Cohen’s *f*^2^ = 0.15) and a significance level of 0.05 to assess whether the sample size was adequate for detecting meaningful effects in the statistical analyses (28). To assess potential clustering by school, we fit a two-level random-intercept linear mixed-effects model and computed the intraclass correlation coefficient (ICC) as the ratio of between-school variance to total variance (29). The impact of loss to follow-up was examined by comparing baseline characteristics of included vs. excluded participants using the standardized mean difference (SMD), reported as absolute values (|SMD|) (30). Differences were considered negligible when |SMD| < 0.10, and larger values were regarded as imbalanced (31). In addition, we quantified differential attrition in key subgroups (age, gender group, and baseline BMI category) by reporting attrition rates and risk differences (RDs) with 95% CIs relative to a prespecified reference level. Descriptive statistics were reported as means ± standard deviation for continuous variables and as counts for categorical variables. To assess regression to the mean (RTM), the change score was computed (Δ = Y_2_ − Y_1_, with Y_1_ = baseline BMI and Y_2_ = follow-up BMI), and a simple linear model was fitted: (Δ = *α* + *ꞵ*Y_1_ + *ε*). A negative *β* indicates that higher baseline values are associated with greater negative change (shrinkage toward the mean). The point estimate of *β*, its 95% confidence interval (CI), and the *p*-value were reported. R^2^ quantified the proportion of variance in Δ attributable to RTM, with a 95% CI obtained via nonparametric bootstrap.

### 3.4 Data preprocessing

This study applied the same data preprocessing to all algorithms to keep an identical feature space. Z-score standardization was applied to numerical features, ordered categorical variables were encoded with prespecified ordinal levels, and unordered categorical variables were represented using one-hot encoding. All preprocessing components were fit on training data (or training folds) only and applied to validation/test sets to prevent leakage.

### 3.5 Model construction

In the development of our prediction model, we adhered to the TRIPOD+AI checklist, and the completed checklist is provided as Supplementary Table 1.

To establish a BMI prediction model, the following regression algorithms were utilized, including CatBoost (CB), LightGBM (LGBM), Neural Network (MLP), Decision Tree (DT), Support Vector Regressor (SVR), and K-Nearest Neighbors (KNN). These models represent a range of learning paradigms, allowing for a comprehensive comparison across different modeling strategies. Among them, tree-based models such as CB and LGBM are especially well-suited for handling high-dimensional, noisy, and heterogeneous health data. First, the dataset was split into training (80%) and independent test (20%) sets, with the test set held out throughout model development and tuning. On the 80% training set, all algorithms underwent 5 × 5 nested cross-validation (CV), with the inner loop performing feature selection and hyperparameter tuning and the outer loop providing unbiased performance estimates (32). Within each inner loop, predictors were prescreened on the inner-training folds using the univariable analyses (Pearson correlation for continuous variables and analysis of covariance (ANCOVA) for categorical variables; *p* < 0.05), followed by five-fold CV with grid search to select the hyperparameter set with the best mean validation score. Features selected across the five inner folds were aggregated by selection frequency to form that outer fold’s consensus feature set (33). In the outer loop, models were retrained on the outer-training folds using the consensus features and inner-optimal hyperparameters, and then evaluated on the outer test folds for unbiased assessment. Model performance was assessed based on four widely used indicators: root mean squared error (RMSE), mean squared error (MSE), mean absolute error (MAE) and coefficient of determination (R^2^). MSE reflects the average squared difference between predicted and actual values, capturing overall model fit. RMSE, the square root of MSE, reports the typical prediction error in the outcome’s original units. MAE provides a direct measure of the average prediction error. R^2^ quantifies the proportion of variance in the outcome that is explained by the model, indicating its explanatory power. After completing all outer folds, performance on the outer test folds was summarized as mean ± SD. The model with the lowest RMSE/MSE/MAE and highest R^2^ was deemed optimal. All outer-fold feature sets were then combined using the same frequency rule to obtain the final feature set. Final hyperparameters were selected via five-fold CV with grid search, then the model was retrained on the entire 80% training set. Complete model-tuning details (hyperparameter search grids, CV folds, seeds, early-stopping settings) are provided in Supplementary Table 2.

### 3.6 Heteroscedasticity investigation and model calibration

Heteroscedasticity was assessed on the 80% training set using out-of-fold residuals via a Breusch–Pagan test (*α* = 0.05) to examine whether prediction errors varied with baseline BMI (34). Results were reported without calibration when the test was not significant, and weighted least-squares (WLS) calibration was applied otherwise (35, 36). Residuals were computed from training out-of-fold predictions and used to fit an empirical variance model to derive sample weights. Using these weights, a linear recalibration of observed versus predicted values was fit on the training data to obtain fixed intercept and slope. The learned weighting function and coefficients were then applied once to the test set without any refitting, avoiding information leakage.

### 3.7 Integrated evaluation of model performance

Overall model performance was compared on the 20% independent test set. First, generalization was assessed with 1,000 bootstrap resamples, reporting RMSE, MSE, MAE, and R^2^ with 95% confidence intervals (CIs) for each model. Paired tests were conducted by bootstrapping the paired differences in RMSE using identical resamples across models (defined as comparator minus best model). This quantified the incremental benefit of the best-performing model, reporting ΔRMSE with its 95% CIs. Second, for the best-performing model, incremental benefit over a trivial baseline (predicting follow-up BMI = baseline BMI) was quantified on the same independent test set using paired bootstrap with 1,000 resamples. Paired differences were defined as ΔRMSE/ΔMSE/ΔMAE = baseline − best and ΔR^2^ = best − baseline, and Δ values were reported with 95% CIs. Third, to assess robustness to a dominant predictor, a sensitivity analysis excluding baseline BMI was performed. Under an identical modeling pipeline to the primary analysis, we retrained and evaluated the model without baseline BMI using the best-performing algorithmic framework, and reported RMSE, MSE, MAE, and R^2^ on the same independent test set.

Stratified performance was evaluated across clinically relevant subgroups, including gender (male vs. female), age groups (14–15 vs. 16–17 years), and baseline BMI category (normal vs. overweight/obesity). For each subgroup, we reported RMSE, MSE, MAE, and R^2^ with 95% CIs. We also computed and reported between-group differences (Δ) in each metric with 95% CIs. Subgroup heterogeneity was assessed using permutation tests, and the significance level was set at 0.05.

Using the same independent test set, overall and stratified error analyses were performed for the calibrated best-performing model. For overall error visualization, we generated predicted-versus-observed scatterplots with a smoothing line and Bland–Altman plot. To characterize the error distribution, we calculated mean error ± SD, mean absolute error (MAD), the interquartile range of |error| (IQR|e|), and the 90th/95th percentiles of |error| (P90|e|/P95|e|), and reported these metrics both overall and stratified by gender, age group, and baseline BMI category.

### 3.8 Model interpretation

This study applied SHAP to provide both global and local interpretation of the best-performing model. We quantified the contribution of each feature to model predictions by examining feature interactions, and decomposed individual predictions into additive feature contributions, using visualizations to convey overall patterns and individual differences. Non-modifiable features were excluded from SHAP visualizations. For global interpretation, we generated a SHAP summary plot based on mean absolute SHAP values across all samples, ranking modifiable predictors for BMI. In addition, we computed SHAP interaction values and displayed a heatmap of their mean strength across samples. SHAP dependence plots were used to examine the overall effect shapes of specific features across the cohort and to highlight potential interactions. For local interpretation, SHAP waterfall plots were generated to break each individual’s predicted BMI into the model’s base value (the average prediction) plus the additive contributions of features, thereby visualizing how specific factors influence the prediction at the individual level.

### 3.9 Reproducibility statement

Analyses were run in Python 3.11.3 (scikit-learn 1.5.2, CatBoost 1.2.7, LightGBM 4.5.0, SHAP 0.46.0). Randomness was controlled by fixing seeds for dataset splitting, outer/inner nested CV splitters, final 5-fold CV, algorithm random seeds. All seed values are listed in Supplementary Table 2. Development was performed in PyCharm 2023.1.3.

## 4 Results

### 4.1 Study characteristics

**Figure 1:**
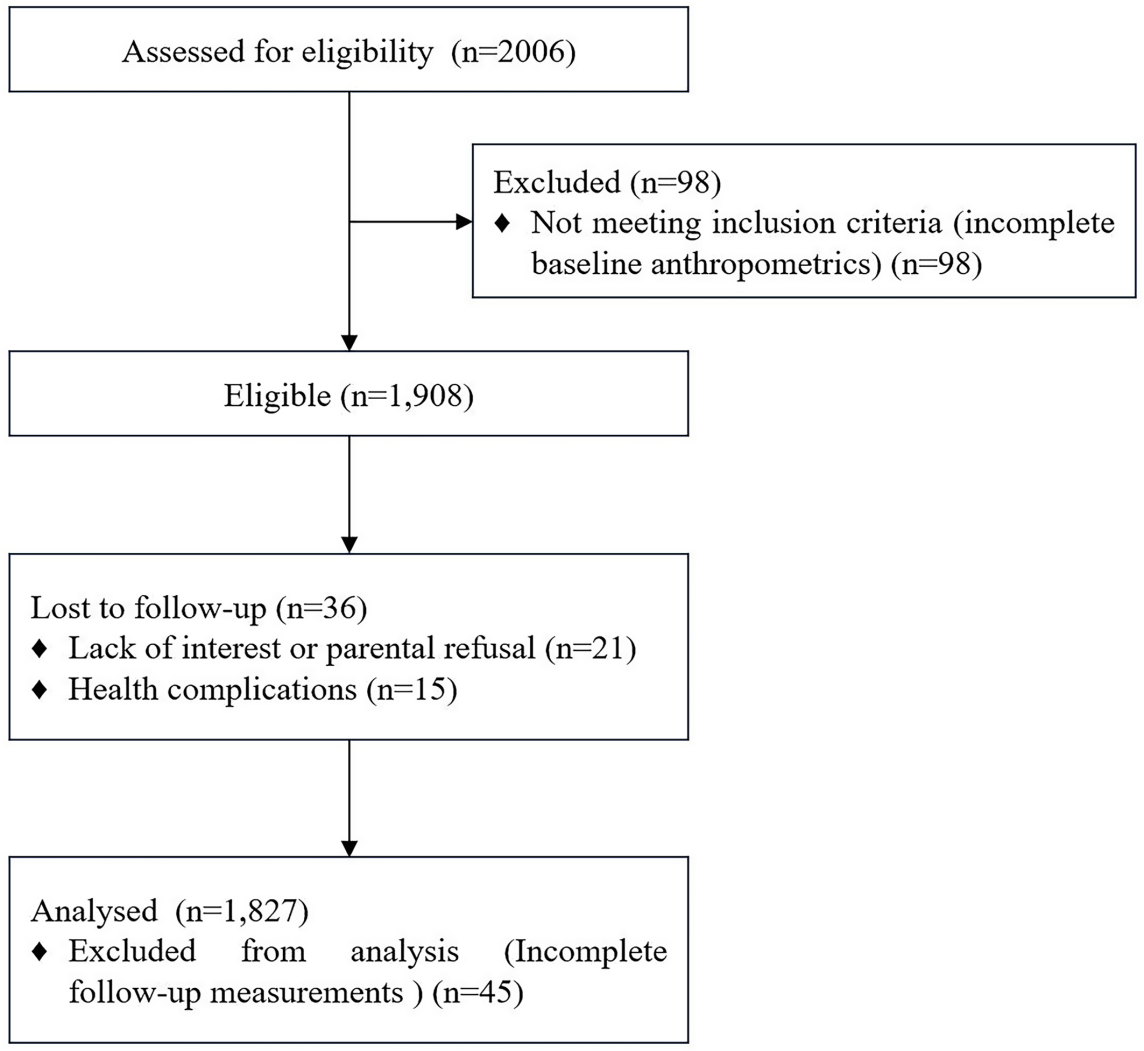
CONSORT flow diagram.

At baseline, a total of 2,006 students aged 14–17 were enrolled in the study. During the one-year follow-up period, 98 students were excluded due to incomplete baseline anthropometrics data, 21 students withdrew due to lack of interest or parental refusal, 45 students were excluded due to incomplete follow-up measurements, and 15 students experienced health complications that hindered their continued participation. Consequently, 1,827 students (1,009 males and 818 females) with complete data at both time points were included in the final analysis. Notably, the electronic questionnaire system ensured all items were completed, guaranteeing no missing values in baseline data. Anthropometric measurements were 100% complete in the final cohort. The participant eligibility, follow-up, and analysis process is illustrated in Figure 1. At baseline, the mean BMI was 21.18 ± 3.63 kg/m^2^, and after 1 year, it increased to 21.54 ± 3.59 kg/m^2^. The mean ΔBMI over the one-year period, calculated as the individual-level difference between follow-up and baseline BMI, was 0.36 ± 1.40 kg/m^2^, reflecting a slight upward trend within the cohort. The power analysis confirmed that the final sample size was sufficient to detect medium-sized effects with adequate statistical power, supporting the validity of the univariate analyses. In addition, the school-level ICC for baseline BMI was approximately zero (95% CI: 0.000–0.0037; *p* > 0.05), indicating negligible clustering. Baseline characteristics are presented in Table 1, comparing included and excluded participants. All |SMD| values were <0.10, indicating good balance of baseline characteristics with negligible differences. The overall loss to follow-up was 4.2%. In prespecified subgroup analyses of attrition rates and RDs versus the reference level, differences were small and imprecise. Age: 14–15 years 4.2% (reference) vs. 16–17 years 4.4%, RD = +0.3 percentage points (95% CI: −1.7–2.2). Gender: female 4.0% (reference) vs. male 4.5%, RD = +0.5 percentage points (95% CI: −1.3–2.3). Baseline BMI category: non-overweight/obese 4.1% (reference) vs. overweight/obese 4.7%, RD = +0.6 percentage points (95% CI: −1.5–2.7). Collectively, subgroup RDs were close to zero and all 95% CIs included zero, providing no evidence of differential attrition. Furthermore, the RTM analysis yielded *β* = −0.084 (95% CI: −0.122 to −0.047; *p* < 0.01), indicating evidence of regression to the mean. The coefficient of determination was R^2^ = 0.051 (95% CI: 0.008–0.119), implying that baseline BMI accounts for about 5% (95% CI: 0.8–11.9%) of the variance in the observed change.

**Table 1:** Baseline characteristics of included and excluded participants.

Baseline characteristic	Included participants (*n* = 1,827)	Excluded participants (*n* = 81)	SMD
Baseline BMI	21.18 ± 3.63	21.14 ± 2.85	0.01
Baseline BMR	1,601.24 ± 223.39	1621.06 ± 250.58	0.08
Age	15.19 ± 1.12	15.21 ± 1.10	0.02
Gender			
Male	1,009 (55.2%)	47 (58.0%)	0.06
Female	818 (44.8%)	34 (42.0%)	0.06
Paternal overweight/obesity status			
Yes	713 (39.0%)	32 (39.5%)	0.01
No	1,114 (61.0%)	49 (60.5%)	0.01
Paternal educational level			
Lower	872 (47.7%)	38 (46.9%)	0.02
Middle	630 (34.5%)	29 (35.8%)	0.03
Higher	325 (17.8%)	14 (17.3%)	0.01
Paternal occupation			
Civil servants and public institutions	210 (11.5%)	10 (12.3%)	0.03
Professional technicians	195 (10.7%)	11 (13.6%)	0.09
Business and service industries	496 (27.1%)	25 (30.9%)	0.08
Workers or farmers	337 (18.4%)	12 (14.8%)	0.09
Homemakers or unemployed	16 (0.9%)	1 (1.2%)	0.04
Others	573 (31.4%)	22 (27.2%)	0.09
Maternal overweight/obesity status			
Yes	358 (19.6%)	15 (18.5%)	0.03
No	1,469 (80.4%)	66 (81.5%)	0.03
Maternal educational level			
Lower	966 (52.9%)	42 (51.9%)	0.02
Middle	637 (34.9%)	27 (33.3%)	0.03
Higher	224 (12.3%)	12 (14.8%)	0.08
Maternal occupation			
Civil servants and public institutions	167 (9.1%)	7 (8.6%)	0.02
Professional technicians	96 (5.3%)	5 (6.2%)	0.04
Business and service industries	449 (24.6%)	20 (24.7%)	0.00
Workers or farmers	248 (13.6%)	11 (13.6%)	0.00
Homemakers or unemployed	311 (17.0%)	14 (17.3%)	0.01
Others	556 (30.4%)	24 (29.6%)	0.02
Family income			
Lowest	514 (28.1%)	23 (28.4%)	0.01
Lower middle	655 (35.9%)	28 (34.6%)	0.03
Upper middle	469 (25.7%)	22 (27.2%)	0.03
Highest	189 (10.3%)	8 (9.9%)	0.02
Family residence location			
Center city	937 (51.3%)	42 (51.9%)	0.01
Non-center city	890 (48.7%)	39 (48.1%)	0.01
On-campus residence			
Yes	1,315 (72.0%)	58 (71.6%)	0.01
No	512 (28.0%)	23 (28.4%)	0.01
Daily sleep duration			
<6 h/day	305 (16.7%)	15 (18.5%)	0.05
6–8 h/day	1,299 (71.1%)	56 (69.1%)	0.04
>8 h/day	233 (12.2%)	10 (12.3%)	0.00
Frequency of staying up late			
Never	370 (20.3%)	16 (19.8%)	0.01
Sometimes	839 (45.9%)	37 (45.7%)	0.00
Often	300 (16.4%)	14 (17.3%)	0.02
Always	318 (17.4%)	14 (17.3%)	0.00
Sedentariness duration on weekends			
3–5 h/day	355 (19.4%)	15 (18.5%)	0.02
5–7 h/day	420 (23.0%)	20 (24.7%)	0.04
7–9 h/day	748 (40.9%)	33 (40.7%)	0.00
>9 h/day	304 (16.6%)	13 (16.0%)	0.02
Schoolwork burden			
Minimal	51 (2.8%)	2 (2.5%)	0.02
Manageable	954 (52.2%)	43 (53.1%)	0.02
High	689 (37.7%)	31 (38.3%)	0.01
Overwhelming	133 (7.3%)	5 (6.2%)	0.04
Frequency of high-protein food intake			
Never	210 (11.5%)	10 (12.3%)	0.03
Sometimes	808 (44.2%)	36 (44.4%)	0.00
Often	463 (25.3%)	20 (24.7%)	0.01
Always	346 (18.9%)	15 (18.5%)	0.01
Frequency of midnight snack intake			
Never	503 (27.5%)	22 (27.2%)	0.01
Sometimes	1,056 (57.8%)	47 (58.0%)	0.00
Often	195 (10.7%)	9 (11.1%)	0.01
Always	73 (4.0%)	3 (3.7%)	0.01
Frequency of high-calorie foods intake			
Never	309 (16.9%)	13 (16.0%)	0.02
Sometimes	916 (50.1%)	41 (50.6%)	0.01
Often	458 (25.1%)	20 (24.7%)	0.01
Always	144 (7.9%)	7 (8.6%)	0.03
Frequency of participation in physical activities			
0 times/week	253 (13.8%)	13 (16.0%)	0.06
1–2 times/week	543 (29.7%)	21 (25.9%)	0.08
2–3 times/week	784 (42.9%)	35 (43.2%)	0.01
> 3 times/week	247 (13.5%)	12 (14.8%)	0.04
Post-exercise sensations			
Relaxed	147 (8.0%)	8 (9.9%)	0.07
Slightly tired	1,103 (60.4%)	49 (60.5%)	0.00
Fairly tired	452 (24.7%)	19 (23.5%)	0.03
Extremely tired	125 (6.8%)	5 (6.2%)	0.03
Physical activities duration on weekends			
<1 h/day	1,130 (61.9%)	49 (60.5%)	0.03
1–2 h/day	517 (28.3%)	24 (29.6%)	0.03
2–3 h/day	101 (5.5%)	4 (4.9%)	0.03
>3 h/day	79 (4.3%)	4 (4.9%)	0.03
Participation in professional sports training			
Yes	207 (11.3%)	11 (13.6%)	0.07
No	1,620 (88.7%)	70 (86.4%)	0.07
Parental support for sports involvement			
Low support	100 (5.5%)	5 (6.2%)	0.03
Moderate support	1,088 (59.6%)	48 (59.3%)	0.01
High support	639 (35.0%)	28 (34.6%)	0.01
Recognize self-weight status correctly			
Yes	1,454 (79.6%)	65 (80.2%)	0.02
No	373 (20.4%)	16 (19.8%)	0.02
Satisfaction with body size			
Yes	770 (42.1%)	35 (43.2%)	0.02
No	1,057 (57.9%)	46 (56.8%)	0.02
Considered changing body size			
Yes	1,184 (64.8%)	54 (66.7%)	0.04
No	643 (35.2%)	27 (33.3%)	0.04
Level of health literacy			
Lowest	345 (18.9%)	15 (18.5%)	0.01
Lower middle	408 (22.3%)	18 (22.2%)	0.00
Upper middle	480 (26.3%)	21 (25.9%)	0.01
Highest	594 (32.5%)	27 (33.3%)	0.02

Values are mean ± SD for continuous variables and n (%) for categorical variables; SMD are reported as absolute value (|SMD|).

### 4.2 Integrated overall model performance

Under nested cross-validation, the CB model performed the best, achieving the lowest RMSE, MSE, and MAE, and the highest R^2^, as shown in Table 2. The final hyperparameters selected for all models are reported in Supplementary Table 2. The final CB model included the following predictors: baseline BMI, baseline BMR, level of health literacy, recognize self-weight status correctly, sedentariness duration on weekends, participation in professional sports training, frequency of staying up late, daily sleep duration, frequency of high-calorie food intake, physical activities duration on weekends, post-exercise sensations, satisfaction with body size, family residence location, and on-campus residence. Table 3 summarizes the performance of all final models on the independent test set, reporting RMSE, MSE, MAE, and R^2^ with 95% CIs. The CB model demonstrated the best generalization. In Addition, paired bootstrap RMSE difference (comparator—CB) for MLP 0.080 (95% CI: 0.012–0.151), LGBM 0.124 (95% CI: 0.065–0.185), SVR 0.076 (95% CI: 0.011–0.142), KNN 0.453 (95% CI: 0.340–0.578), and DT 0.171 (95% CI: 0.074–0.268), with all intervals strictly positive, confirming lower RMSE for CB and its significant superiority over the comparator models.

**Table 2:** The performance of each algorithm in terms of RMSE, MSE, MAE, and R^2^ on nested cross-validation.

Model	RMSE (mean ± SD)	MSE (mean ± SD)	MAE (mean ± SD)	R^2^ (mean ± SD)
CatBoost	1.204 **±** 0.063	1.453 **±** 0.152	0.900 **±** 0.043	0.882 **±** 0.013
LightGBM	1.288 **±** 0.034	1.659 **±** 0.087	0.969 **±** 0.029	0.864 **±** 0.018
Neural Network	1.233 **±** 0.080	1.526 **±** 0.192	0.935 **±** 0.059	0.875 **±** 0.020
Decision Tree	1.423 **±** 0.091	2.032 **±** 0.258	1.034 **±** 0.047	0.833 **±** 0.035
Support Vector Regressor	1.247 **±** 0.059	1.558 **±** 0.145	0.918 **±** 0.041	0.873 **±** 0.014
K-Nearest Neighbors	1.617 **±** 0.052	2.618 **±** 0.167	1.251 **±** 0.044	0.787 **±** 0.022

RMSE, root mean squared error; MSE, mean squared Error; MAE, mean absolute error; R^2^, coefficient of determination; CI, confidence interval.

**Table 3:** The performance of each algorithm in terms of MSE, RMSE, MAE, and R^2^ on the independent test set.

Model	RMSE (95% CI)	MSE (95% CI)	MAE (95% CI)	R^2^ (95% CI)
CatBoost	1.212 (1.113–1.322)	1.468 (1.238–1.748)	0.897 (0.820–0.987)	0.900 (0.878–0.917)
LightGBM	1.336 (1.225–1.451)	1.784 (1.501–2.107)	0.990 (0.902–1.085)	0.879 (0.852–0.899)
Neural Network	1.293 (1.174–1.413)	1.671 (1.377–1.995)	0.965 (0.879–1.051)	0.886 (0.862–0.907)
Decision Tree	1.381 (1.256–1.516)	1.908 (1.576–2.299)	0.981 (0.890–1.083)	0.870 (0.842–0.893)
Support Vector Regressor	1.288 (1.165–1.419)	1.658 (1.357–2.014)	0.935 (0.849–1.030)	0.887 (0.861–0.909)
K-Nearest Neighbors	1.667 (1.513–1.820)	2.778 (2.288–3.311)	1.274 (1.165–1.385)	0.811 (0.780–0.839)

RMSE, root mean squared error; MSE, mean squared Error; MAE, mean absolute error; R^2^, coefficient of determination; CI, confidence interval.

The Breusch–Pagan test on training out-of-fold residuals indicated heteroscedasticity of errors with baseline BMI (*p* < 0.05), so we applied WLS calibration learned on the training data and then applied to the test set without refitting. After calibration, test-set performance was: RMSE 1.200 (95% CI: 1.101–1.303), MSE 1.440 (95% CI: 1.211–1.697), MAE 0.895 (95% CI: 0.818–0.981) and R^2^ 0.902 (95% CI: 0.882–0.918).

As a trivial baseline model (predicting follow-up BMI equals baseline BMI), performance on the independent test set was MSE 2.212 (95% CI: 1.766–2.767), RMSE 1.487 (95% CI: 1.329–1.663), MAE 1.065 (95% CI: 0.966–1.181) and R^2^ 0.850 (95% CI: 0.808–0.883). Using bootstrap of paired differences, the incremental performance benefit of the CB-based model over the trivial baseline model was: ΔRMSE (baseline − CB) 0.276 (95% CI: 0.159–0.389), ΔMSE 0.752 (95% CI: 0.415–1.115), ΔMAE 0.169 (95% CI: 0.093–0.242) and ΔR^2^ (CB − baseline) 0.051 (95% CI: 0.027–0.078). All intervals exclude zero, indicating that the CB model provides a statistically significant improvement over the baseline.

After excluding baseline BMI, the CB model achieved RMSE 2.497 (95%: CI 2.270–2.728), MSE 6.233 (95% CI: 5.154–7.440), MAE 1.877 (95% CI: 1.709–2.045), and R^2^ 0.576 (95% CI: 0.502–0.644) on the independent test set. Compared with the primary model, performance declined, suggesting that baseline BMI is likely a dominant predictor.

### 4.3 Stratified performance and error distribution

In subgroup performance evaluation, Table 4 presents the stratified metrics (RMSE, MSE, MAE, R^2^) of the calibrated CB model, with paired-bootstrap between-group differences (Δ with 95% CIs). In permutation tests, *p*-values for all performance metrics by gender and by age group were >0.05. For baseline BMI categories, *p* < 0.05. These results indicate that performance differences across gender and age groups were small, with no statistically detectable heterogeneity. Statistically significant differences were observed across baseline BMI categories.

**Table 4:** Stratified model performance and between-group differences.

Feature	Group	RMSE (95% CI)	MSE (95% CI)	MAE (95% CI)	R^2^(95% CI)
Gender	Male	1.197 (1.065–1.332)	1.433 (1.135–1.775)	0.898 (0.794–1.015)	0.903 (0.876–0.925)
	Female	1.217 (1.050–1.380)	1.480 (1.102–1.906)	0.895 (0.771–1.027)	0.899 (0.866–0.923)
	Δ (female–male)	0.088 (0.004–0.239)	0.212 (0.009–0.601)	0.067 (0.002–0.189)	0.015 (0.001–0.044)
Age	14–15 y	1.226 (1.098–1.349)	1.502 (1.206–1.819)	0.918 (0.818–1.018)	0.899 (0.875–0.918)
	16–17 y	1.169 (0.982–1.343)	1.366 (0.965–1.805)	0.860 (0.728–1.008)	0.904 (0.863–0.931)
	Δ (16–17−14–15)	0.102 (0.003–0.273)	0.243 (0.008–0.653)	0.085 (0.004–0.226)	0.016 (0.001–0.044)
Baseline BMI	Normal	0.979 (0.878–1.079)	0.959 (0.770–1.165)	0.734 (0.661–0.810)	0.806 (0.748–0.847)
	Overweight/obesity	1.658 (1.460–1.862)	2.748 (2.130–3.469)	1.324 (1.147–1.524)	0.670 (0.550–0.739)
	Δ (OW/OB − Normal)	0.682 (0.473–0.895)	1.811 (1.171–2.483)	0.597 (0.407–0.790)	0.142 (0.044–0.251)

RMSE, root mean squared error; MSE, mean squared Error; MAE, mean absolute error; R^2^, coefficient of determination; CI, confidence interval; Δ, between-group difference.

In error distribution analysis, the calibrated CB model showed near-zero overall bias, mean error = 0.03 ± 1.21, with central and tail dispersion MAD = 0.66, IQR|e| = 1.05, P90|e| = 1.98, P95|e| = 2.71. By gender, males (−0.03 ± 1.20) and females (0.10 ± 1.22) were similar, indicating no material heterogeneity by gender. By age, 14–15 years (−0.01 ± 1.23) and 16–17 years (0.08 ± 1.17) were only mildly different, indicating no material heterogeneity by age. By baseline BMI category, errors in the overweight/obesity group (0.24 ± 1.65) were larger and more right-shifted than in the normal group (−0.06 ± 0.98).

**Figure 2:**
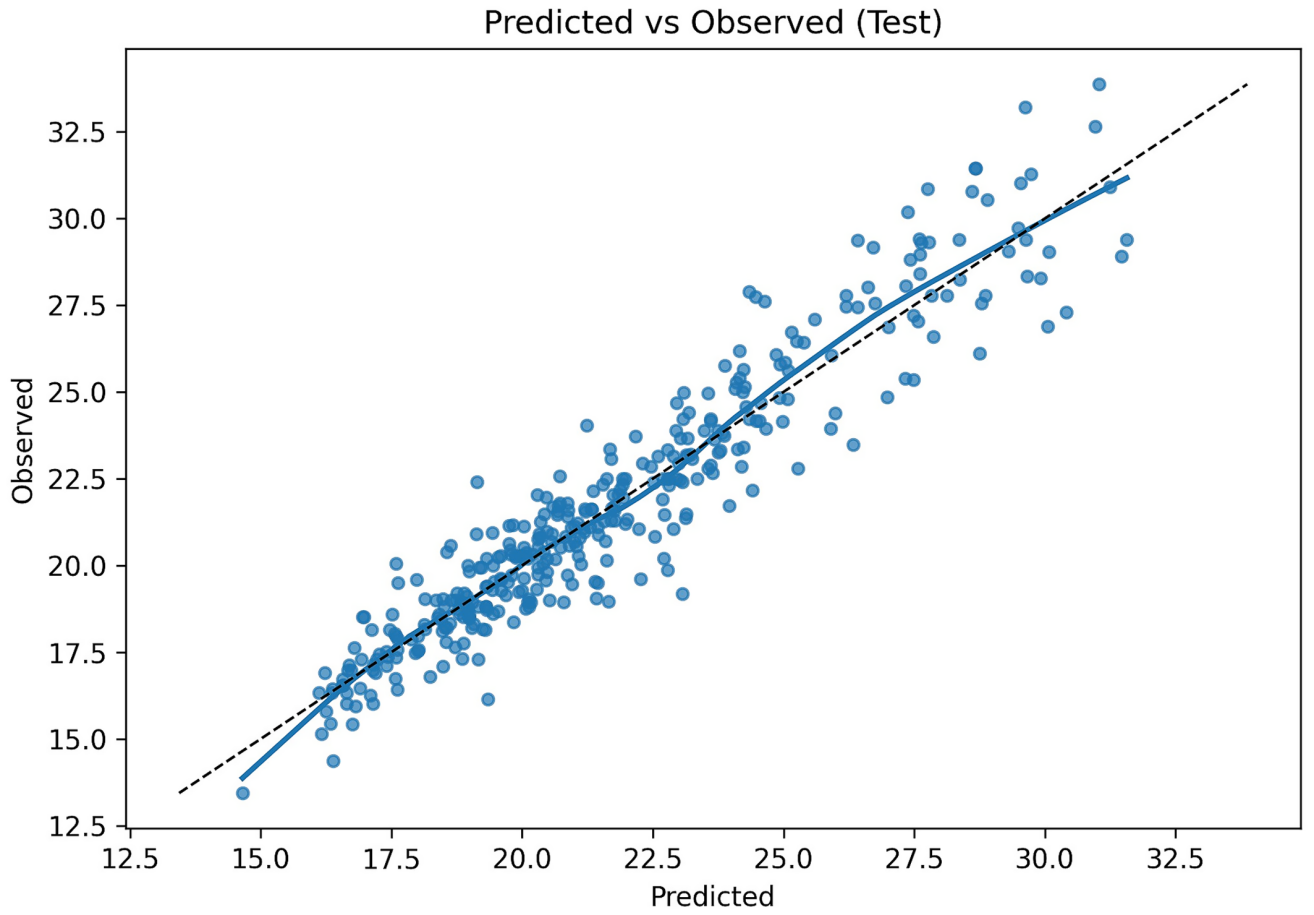
Predicted-versus-observed scatter plot.

**Figure 3:**
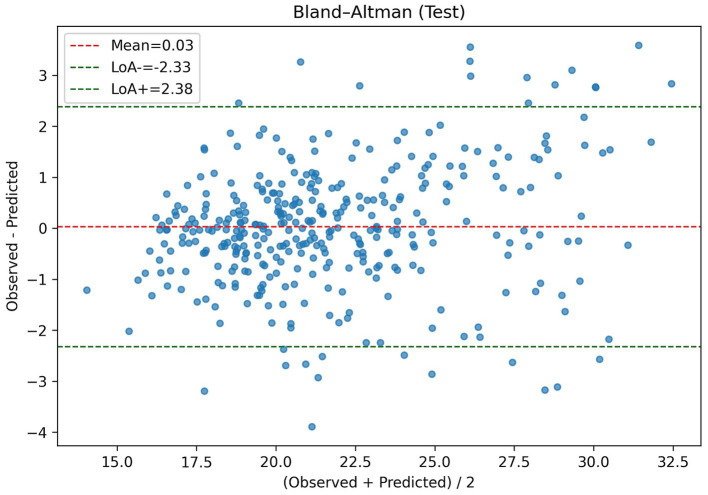
Bland–Altman plot.

For overall error visualization, Figure 2 shows the predicted-versus-observed scatter with a smoothing line, indicating overall fit with only slight departures at the extremes of the prediction range. Figure 3 displays the Bland–Altman plot with a near-zero mean bias and approximately symmetric limits of agreement (−2.33 to 2.38), and the data points show no systematic drift with the mean, suggesting negligible bias.

### 4.4 Global interpretability

**Figure 4:**
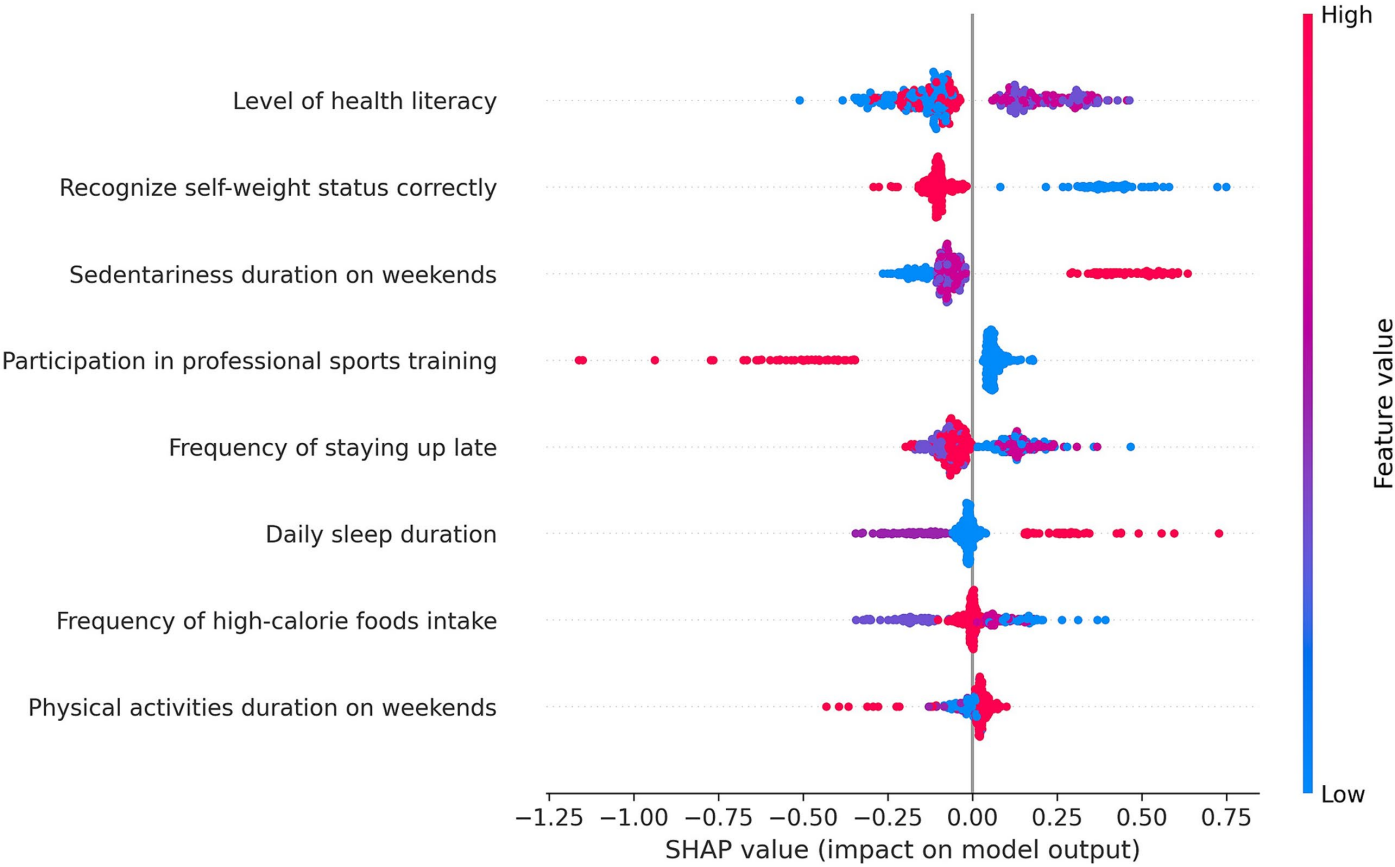
SHAP summary plot for feature importance. Each point represents a sample’s SHAP value for a feature. Color indicates the value of the feature (red = high, blue = low). Features are ranked by their mean absolute SHAP values, reflecting their overall contribution to BMI prediction.

Population-level feature importance was assessed using SHAP global interpretation applied to the CB-based model. To focus on modifiable features for intervention insights, non-modifiable features such as baseline BMI, gender, family residence location, among others, were excluded from SHAP visual analysis. Figure 4 displays the SHAP summary plot, ranking modifiable features by their mean absolute SHAP values, which represent their average contribution to BMI prediction across the entire population. The plot also visualizes the distribution of SHAP values, with feature color indicating feature values (red: high, blue: low). Features with positive SHAP values contribute positively to BMI prediction, while negative values indicate a decreasing effect. The modifiable features ranked in descending order were: level of health literacy, recognize self-weight status correctly, sedentariness duration on weekends, participation in professional sports training, frequency of staying up late, daily sleep duration, frequency of high-calorie foods intake, physical activities duration on weekends.

**Figure 5:**
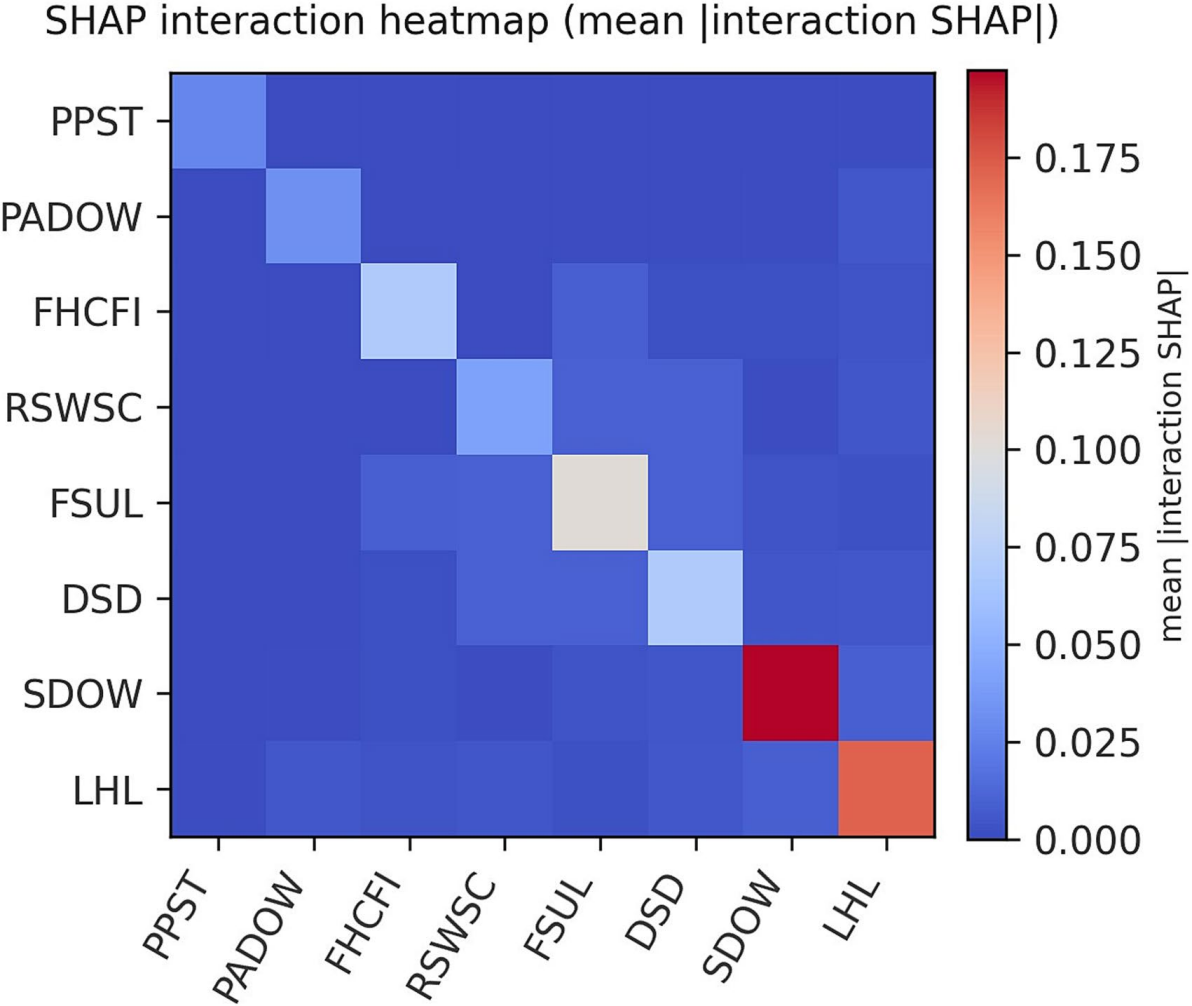
Heatmap of SHAP interaction values. Color intensity encodes interaction strength, with darker/warmer colors indicating stronger interactions and lighter/cooler colors indicating weaker ones. LHL, Level of health literacy; SDOW, Sedentariness duration on weekends; FSUL, Frequency of staying up late; DSD, Daily sleep duration; RSWSC, Recognize self-weight status correctly; FHCFI, Frequency of high-calorie foods intake; PADOW, Physical activities duration on weekends; PPST, Participation in professional sports training.

The SHAP interaction analyses are presented in Figure 5. The heatmap shows the mean absolute SHAP interaction value for each feature pair. Color intensity encodes interaction strength, with darker/warmer colors indicating stronger interactions and lighter/cooler colors indicating weaker ones. Diagonal cells approximate main effects, while off-diagonal cells reflect pairwise interactions. As shown in Figure 5, most feature pairs exhibit near-zero interaction values, indicating predominance of main effects and no strong pairwise interactions.

**Figure 6:**
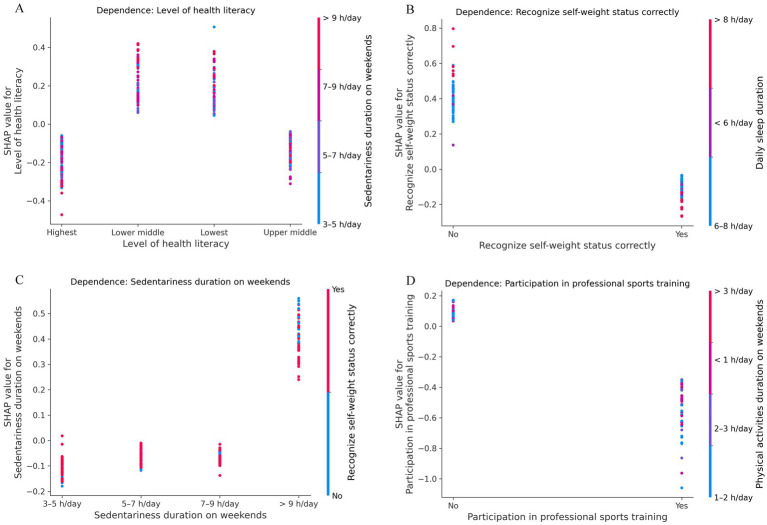
SHAP dependence plots. **(A)** Level of health literacy. **(B)** Recognize self-weight status correctly. **(C)** Sedentariness duration on weekends. **(D)** Participation in professional sports training. The x-axis shows feature values and the y-axis shows SHAP values. Point color indicates the strongest interacting feature, and vertical dispersion reflects potential interactions.

Figure 6 shows SHAP dependence plots for the top four features ranked by global importance (mean |SHAP|). The x-axis shows feature values and the y-axis shows SHAP values. Point color indicates the strongest interacting feature, and vertical dispersion reflects potential interactions. Level of health literacy was positive for “lowest/lower-middle” and negative for “upper-middle/highest” (Figure 6A). Recognize self-weight status correctly was negative for “Yes” and positive for “No” (Figure 6B). Sedentariness duration on weekends >9 h/day showed the largest positive SHAP values, whereas 3–9 h/day was negative to mildly negative (Figure 6C). Participation in professional sports training was negative for “Yes” and near-zero to mildly positive for “No” (Figure 6D). Overall, the plots show smooth directional trends with limited vertical spread, indicating predominance of main effects and modest interactions.

### 4.5 Local interpretability

**Figure 7:**
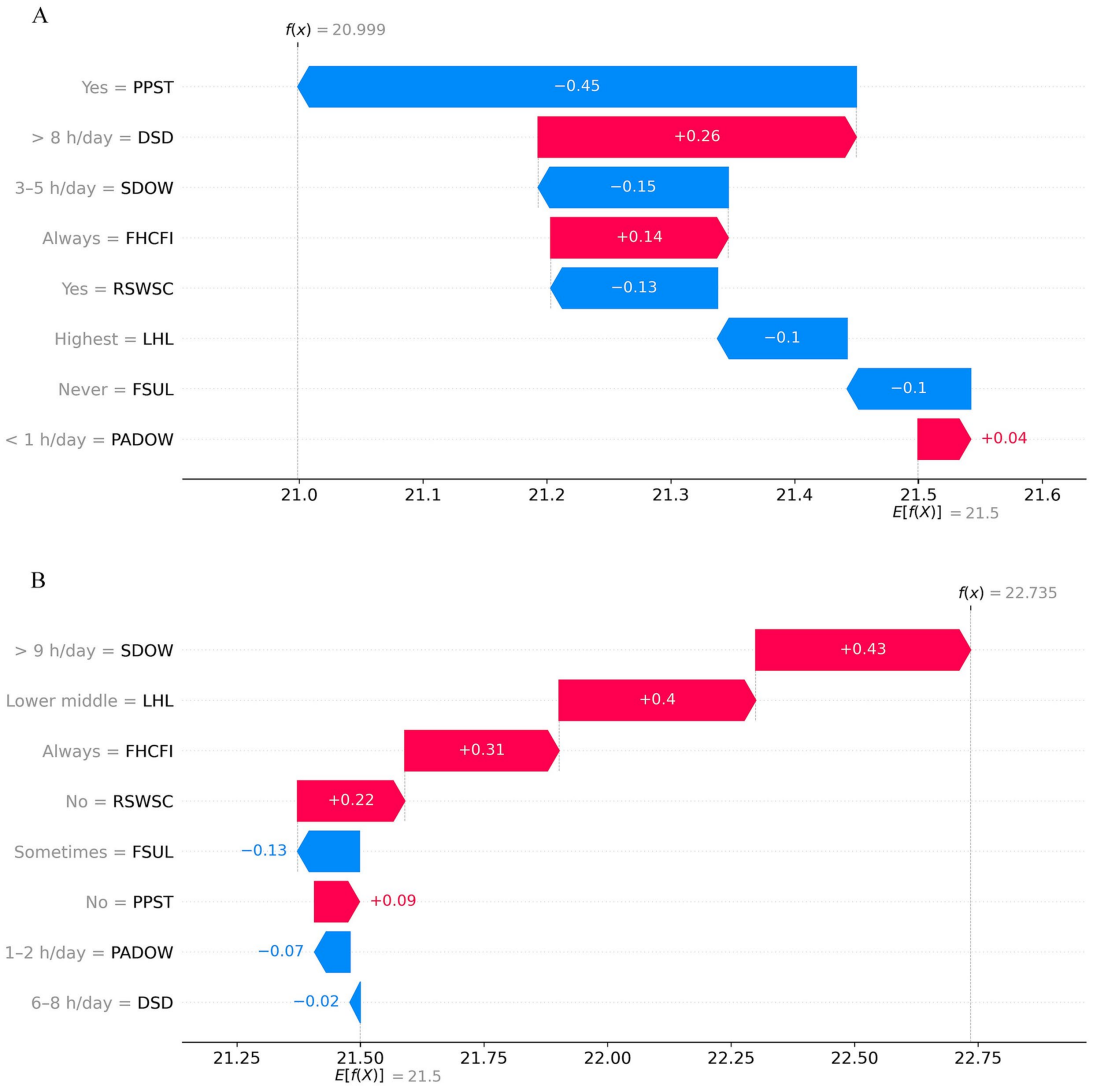
SHAP waterfall plots. The influence of features on BMI prediction for two samples: **(A)** shows a sample with a decrease, and **(B)** shows a sample with an increase in BMI. The red bars indicate positive contributions (increasing the predicted BMI) and the blue bars indicate negative contributions (decreasing the predicted BMI). The bar length and its numeric label reflect the effect magnitude in kg/m^2^, and longer bars denote larger increases or decreases in the predicted BMI. LHL, Level of health literacy; SDOW, Sedentariness duration on weekends; FSUL, Frequency of staying up late; DSD, Daily sleep duration; RSWSC, Recognize self-weight status correctly; FHCFI, Frequency of high-calorie foods intake; PADOW, Physical activities duration on weekends; PPST, Participation in professional sports training.

SHAP values quantify each feature’s association with the model’s prediction, providing a detailed view of how individual modifiable features relate to the model’s predicted BMI values for specific samples. SHAP waterfall plots for two specific samples are shown in Figure 7. The red bars represent positive contributions, indicating an increase in the predicted outcome, while the blue bars represent negative contributions, indicating a decrease in the predicted outcome. The red bars indicate positive contributions (increasing the predicted BMI) and the blue bars indicate negative contributions (decreasing the predicted BMI). The bar length and its numeric label reflect the effect magnitude in kg/m^2^, and longer bars denote larger increases or decreases in the predicted BMI. As shown in Figure 7A, the predicted BMI for this individual is 21.00 kg/m^2^. Negative contributions dominate and reduce the overall prediction. The main negative features and their numerical contributions to the predicted BMI are: PPST (Participation in professional sports training) = Yes (−0.45 kg/m^2^), SDOW (Sedentariness duration on weekends) = 3–5 h/day (−0.15 kg/m^2^), and RSWSC (Recognize self-weight status correctly) = Yes (−0.13 kg/m^2^). Positive contributions are smaller, including DSD (Daily sleep duration) = > 8 h (+0.26 kg/m^2^) and FHCFI (Frequency of high-calorie foods intake) = Always (+0.14 kg/m^2^). In Figure 7B, positive contributions predominate, leading to a higher predicted BMI for this individual.

## 5 Discussion

This study investigated the factors influencing BMI changes in adolescents and conducted a one-year longitudinal cohort study to examine the changes in BMI over this period. Subsequently, a BMI prediction model was developed and validated using machine learning algorithms. Finally, the SHAP model interpretation technique was employed to explore the impact of modifiable factors on BMI changes in adolescents.

In this study, predictors were prescreened on the inner-training folds using the univariable analyses. The statistically significant associations observed suggest that these factors may play an important role in BMI changes among adolescents. Specifically, we found that among adolescents, poor sleep quality, sedentary behavior, and unhealthy eating habits were associated with higher model-predicted BMI, while regular physical activity helps reduce them. In addition, our study shows that adolescents who correctly recognize their weight status, as well as those dissatisfied with their body shape and willing to change, are more likely to drive BMI changes through active intervention. Therefore, self-perception factors play a crucial role in BMI changes, particularly when adolescents recognize their weight issues, which makes them more likely to take proactive steps to manage their weight. Moreover, greater health literacy was also associated with better weight control by enabling adolescents to make informed dietary and lifestyle choices, leading to more stable BMI changes. These findings are consistent with previous research (8, 9, 26, 37, 38).

The CB regression algorithm demonstrated superior performance among the developed BMI prediction models, exceeding results reported in previous research (10, 12, 14, 22, 39–41), as summarized in Table 5. Our model’s strong performance reflects both algorithmic and methodological choices. Specifically, CB’s ordered boosting suppresses target leakage during training and reduces prediction shift. In particular, its symmetric tree structure helps control variance, enhances stability, and mitigates overfitting. Furthermore, CB captures nonlinear relationships effectively under mixed feature types and moderate sample sizes, making it well suited to BMI prediction tasks that involve diverse health data. For model training, this study employed nested CV and achieved strong performance on an independent test set, supporting the model’s generalizability. Nested CV separates hyperparameter tuning from performance assessment, reducing optimistic bias and guarding against information leakage. Despite the use of nested cross-validation and evaluation on an independent test set supporting generalizability, real-world deployment still requires external validation across heterogeneous populations. Because sociocultural norms, dietary patterns, and environmental factors may influence participants’ lifestyle behaviors, body composition, and health awareness, which in turn affect key predictive features of the model, future research will include external validation across more diverse populations to enhance its generalizability. In addition, in the comparison between the CB model and the trivial baseline model, all performance metrics showed significant improvements. The results indicate that the CB model clearly outperforms the baseline model, highlighting the added value of machine learning algorithms in predicting BMI. Moreover, in the sensitivity analysis excluding baseline BMI, all performance metrics of the model declined, indicating that baseline BMI is a key predictor in our model. The importance of baseline BMI provides a foundation for future model improvements.

**Table 5:** Comparison of BMI prediction models in previous studies.

Author name	Features	Method	Main model performance
Park et al. (10)	Neuroimaging features	Machine learning approaches	RMSE = 1.29
Yao et al. (12)	Smartphone motion sensor data	Hybrid deep neural network	MAEs = 2.461 ± 1.000 at MobiAct dataset, 3.137 ± 1.300 at Motion-Sense dataset
Ali et al. (14)	Medication data	Gradient-boosted machine (GBM) learning	RMSE = 4.97
Singh and Tawfik (22)	Earlier BMI values	Regression methods, artificial neural network	MAE = 1.42
Harrison et al. (39)	Clinical, genetic and expression data	Eleven standard regression methods	*R*^2^ = 0.829, RMSE = 2.84 (Rank 1)
Cheng et al. (40)	Early-life EHR data	Support vector regression	MAE = 0.96 at 30–36 months, 0.98 at 36–42 months, and 1.00 at 42–48 months
Delnevo et al. (41)	Psychological variables	Eight machine learning algorithms	MAE = 5.27–5.50

In this study, we employed SHAP techniques to interpret the BMI prediction model, providing both global and local model explanations. Recent studies have widely applied SHAP in various domains, including medicine (42, 43), materials science (44), transportation (45), and others. Compared to methods such as Local Interpretable Model-agnostic Explanation (LIME) and Partial Dependence Plot (PDP), which are commonly used in other studies (46, 47), SHAP offers a more unified and comprehensive framework. In our study, SHAP analysis focused exclusively on modifiable features, such as physical activity, diet, and lifestyle habits, providing both global and local explanations for BMI predictions, enhancing model transparency and credibility, and offering insights to inform personalized interventions.

In the global explanation, bee plots were used to visualize the importance of modifiable features and their overall contribution to the model’s BMI predictions across the dataset. This population-level interpretation provides actionable insights for public health policy development in school and community settings, as it highlights which modifiable behaviors are most strongly associated with the model’s BMI predictions among adolescents. For instance, policies aimed at integrating health literacy education into school curricula may empower adolescents to make healthier lifestyle choices. School- and community-based health literacy campaigns can be implemented to educate adolescents about nutrition, physical activity, and the health consequences of obesity. Weekend community sports programs and family-oriented outdoor activities should be promoted to reduce sedentary behavior. Policy measures may also be considered to reduce excessive academic pressure that may contribute to sleep deprivation. Moreover, population-level feature importance rankings can help policymakers prioritize resource allocation, thereby enhancing the efficiency of public health agencies in implementing adolescent weight-related prevention and intervention programs. Developing interventions targeting top-ranked factors is likely to yield more substantial population-level health benefits than focusing on lower-ranked ones. In addition, interaction heatmaps and dependence plots help guide actions based on model results. Strong interactions call for paired joint interventions, whereas weak interactions with directional dependence curves support targeting single behaviors.

In the local explanation, waterfall plots were generated to decompose each individual prediction into the model’s base value and the numeric contribution of each feature in kg/m^2^, providing clinically interpretable effect sizes. For example, the sample shown in Figure 7B demonstrates a significant risk of BMI increase over the next year if current lifestyle habits are maintained. The SHAP waterfall plot highlights several key risk factors, such as very long weekend sedentary time (SDOW = > 9 h/day, +0.43 kg/m^2^), lower health literacy (LHL = lower middle, +0.40 kg/m^2^), always consuming high-calorie foods (FHCFI = always, +0.31 kg/m^2^), and not recognizing self-weight status correctly (RSWSC = no, +0.22 kg/m^2^). Additionally, although this sample exhibits some positive lifestyle habits, their contributions to the prediction are relatively small, including sometimes staying up late (−0.13 kg/m^2^), 1–2 h/day of weekend physical activity (−0.07 kg/m^2^), and 6–8 h/day of sleep (−0.02 kg/m^2^). This individual-level interpretability analysis helps characterize risk and protective factors associated with variation in the model’s predicted risk of abnormal BMI. Based on the model outputs, clinicians and health management professionals can refer to these findings to design more targeted, personalized intervention strategies to help individuals modify or maintain their current lifestyle habits, which may enhance the precision and effectiveness of adolescent weight management.

To address practical challenges in potential clinical implementation, our study highlights several exploratory advantages that may facilitate future use, pending external validation and impact assessment. First, compared to prediction models that rely on complex and hard-to-obtain medical data (10, 12, 14), the predictors used in this study were derived from easily accessible questionnaire data. This approach reduces the burden of data collection and the cost without compromising model performance, providing an efficient solution for screening high-risk adolescents. Second, predicting continuous BMI values may be useful for quantifying subtle weight changes, potentially aiding dynamic health monitoring about risk trends near commonly thresholds. In contrast, models that use BMI categories as prediction targets (11, 15, 48, 49) are limited to reflecting coarse changes in weight status and may miss critical early warning signs. Third, the integration of SHAP-based interpretability may improve transparency relative to machine-learning black-box models, highlighting how modifiable factors are associated with the model’s predictions of BMI.

The study has the following limitations: (1) The sample was limited in scope, which may affect the generalizability of the findings. Although loss-to-follow-up analyses suggested no strong differential attrition, selection processes and exclusions may still introduce bias. Future research should include participants from different cultural backgrounds, regions, and age groups to enhance model applicability, and should incorporate strategies to mitigate selection and attrition bias. (2) The behavioral habits assessed at baseline may change over the follow-up period. Future studies should consider incorporating dynamic assessments of these behaviors over time to better capture their impact on BMI changes. (3) All questionnaire data were self-reported, which may lead to potential reporting bias. (4) The model has not yet undergone external or temporal validation. While independent datasets yielded strong performance, future work will test the model across external datasets and time points to ensure generalizability. (5) Periodic model updates will be needed to maintain long-term applicability. (6) Pubertal maturation (e.g., Tanner staging) was not collected.

## 6 Conclusion

This study successfully developed a machine learning predictive model for BMI in adolescents based on readily accessible daily information, achieving high predictive performance. The integration of SHAP for model interpretation provided valuable insights into the key factors associated with the model’s predictions of BMI variation. The findings can provide valuable data to inform the formulation of public health policies and may support health-status monitoring while informing the design of personalized intervention strategies for weight and health management.

## Data Availability

The raw data supporting the conclusions of this article will be made available by the authors, without undue reservation.

## References

[1] Khanna D Peltzer C Kahar P Parmar MS. Body mass index (BMI): a screening tool analysis. Cureus. (2022) 14:e22119. doi: 10.7759/cureus.22119, PMID: 35308730 PMC8920809

[2] Wada K Kuboyama K Abe SK Rahman MS Islam MR Saito E . Body mass index and breast cancer risk in premenopausal and postmenopausal east Asian women: a pooled analysis of 13 cohort studies. Breast Cancer Res. (2024) 26:158. doi: 10.1186/s13058-024-01907-5, PMID: 39543702 PMC11566150

[3] Samson R Ennezat PV Le Jemtel TH Oparil S. Cardiovascular disease risk reduction and body mass index. Curr Hypertens Rep. (2022) 24:535–46. doi: 10.1007/s11906-022-01213-5, PMID: 35788967

[4] Ozawa H Fukui K Fujita Y Ishibashi C Yoneda S Nammo T . Expansion of human alpha-cell area is associated with a higher maximum body mass index before the onset of type 2 diabetes. J Diabetes. (2023) 15:277–82. doi: 10.1111/1753-0407.13370, PMID: 36843206 PMC10036255

[5] Seo JY Jin EH Chung GE Kim YS Bae JH Yim JY . The risk of colorectal cancer according to obesity status at four-year intervals: a nationwide population-based cohort study. Sci Rep. (2023) 13:8928. doi: 10.1038/s41598-023-36111-6, PMID: 37264099 PMC10235025

[6] World Obesity Federation. World obesity atlas 2024, (2024). Available online at: https://data.worldobesity.org/publications/?cat=22 (Accessed October 24, 2025).

[7] Silventoinen K Jelenkovic A Sund R Hur YM Yokoyama Y Honda C . Genetic and environmental effects on body mass index from infancy to the onset of adulthood: an individual-based pooled analysis of 45 twin cohorts participating in the COllaborative project of development of anthropometrical measures in twins (CODATwins) study. Am J Clin Nutr. (2016) 104:371–9. doi: 10.3945/ajcn.116.130252, PMID: 27413137 PMC4962159

[8] Zink J Booker R Wolff-Hughes DL Allen NB Carnethon MR Alexandria SJ . Longitudinal associations of screen time, physical activity, and sleep duration with body mass index in US youth. Int J Behav Nutr Phys Act. (2024) 21:35. doi: 10.1186/s12966-024-01587-6, PMID: 38566134 PMC10988901

[9] Sandri E Piredda M Sguanci M Mancin S. What factors influence obesity in Spain? A multivariate analysis of sociodemographic, nutritional, and lifestyle factors affecting body mass index in the Spanish population. Healthcare. (2025) 13:386. doi: 10.3390/healthcare13040386, PMID: 39997261 PMC11855512

[10] Park BY Chung CS Lee MJ Park H. Accurate neuroimaging biomarkers to predict body mass index in adolescents: a longitudinal study. Brain Imaging Behav. (2020) 14:1682–95. doi: 10.1007/s11682-019-00101-y, PMID: 31065926

[11] Gozukara Bag HG Yagin FH Gormez Y González PP Colak C Gülü M . Estimation of obesity levels through the proposed predictive approach based on physical activity and nutritional habits. Diagnostics. (2023) 13:2949. doi: 10.3390/diagnostics13182949, PMID: 37761316 PMC10529319

[12] Yao Y Song L Ye J. Motion-to-BMI: using motion sensors to predict the body mass index of smartphone users. Sensors. (2020) 20:1134. doi: 10.3390/s20041134, PMID: 32093013 PMC7070876

[13] Kim S Lee K Lee EC. Multi-view body image-based prediction of body mass index and various body part sizes. 2023 IEEE/CVF Conference on Computer Vision and Pattern Recognition Workshops (CVPRW). Piscataway, NJ, USA: IEEE. (2023) 6034–6041.

[14] Ali S Na R Waterhouse M Jordan SJ Olsen CM Whiteman DC . Predicting obesity and smoking using medication data: a machine-learning approach. Pharmacoepidemiol Drug Saf. (2022) 31:91–9. doi: 10.1002/pds.5367, PMID: 34611961

[15] Helforoush Z Sayyad H. Prediction and classification of obesity risk based on a hybrid metaheuristic machine learning approach. Front Big Data. (2024) 7:1469981. doi: 10.3389/fdata.2024.1469981, PMID: 39403430 PMC11471553

[16] Alkhanbouli R Matar Abdulla Almadhaani H Alhosani F Simsekler MCE. The role of explainable artificial intelligence in disease prediction: a systematic literature review and future research directions. BMC Med Inform Decis Mak. (2025) 25:110. doi: 10.1186/s12911-025-02944-6, PMID: 40038704 PMC11877768

[17] Lundberg SM Lee S-I. A unified approach to interpreting model predictions. Proceedings of the 31st International Conference on Neural Information Processing Systems. Red Hook, NY, USA: Curran Associates, Inc. (2017) 4768–4777.

[18] Li X Li L Zhang L. Development and validation of a prediction model for myelosuppression in lung cancer patients after platinum-based doublet chemotherapy: a multifactorial analysis approach. Am J Cancer Res. (2025) 15:470–86. doi: 10.62347/TFUC2568, PMID: 40084374 PMC11897629

[19] Luo XQ Kang YX Duan SB Yan P Song GB Zhang NY . Machine learning-based prediction of acute kidney injury following pediatric cardiac surgery: model development and validation study. J Med Internet Res. (2023) 25:e41142. doi: 10.2196/41142, PMID: 36603200 PMC9893730

[20] Yu L Cao S Song B Hu Y. Predicting grip strength-related frailty in middle-aged and older Chinese adults using interpretable machine learning models: a prospective cohort study. Front Public Health. (2024) 12:1489848. doi: 10.3389/fpubh.2024.1489848, PMID: 39741944 PMC11685125

[21] Arumäe K Mõttus R Vainik U. Body mass predicts personality development across 18 years in middle to older adulthood. J Pers. (2023) 91:1395–409. doi: 10.1111/jopy.12816, PMID: 36718127

[22] Singh B Tawfik H. A machine learning approach for predicting weight gain risks in young adults. 2019 10th International Conference on Dependable Systems, Services and Technologies (DESSERT). Piscataway, NJ, USA: IEEE. (2019) 231–234.

[23] FAO/WHO/UNU. Annex 1: equations for the prediction of basal metabolic rate. (2004). Available online at: https://www.fao.org/4/aa040e/AA040E15.htm (Accessed October 24, 2025).

[24] FAO/WHO/UNU Expert Consultation. (2004). Human energy requirements. Rome: Food and Agriculture Organization of the United Nations. Available online at: https://openknowledge.fao.org/handle/20.500.14283/y5686e (Accessed October 24, 2025).

[25] Group of China Obesity Task F. Body mass index reference norm for screening overweight and obesity in Chinese children and adolescents. Zhonghua Liu Xing Bing Xue Za Zhi. (2004) 25:97–102. doi: 10.3760/j.issn:0254-6450.2004.02.003 PMID: 15132858

[26] Ramirez Luque DB Rocha Huaman NL Calizaya-Milla YE Calizaya-Milla SE Ramos-Vera C Saintila J. Body self-perception, dietary self-efficacy, and body mass index in young adults: a cross-sectional survey. Int J Gen Med. (2023) 16:193–202. doi: 10.2147/IJGM.S395281, PMID: 36699341 PMC9869799

[27] Li C Zhang M Tarken AY Cao Y Li Q Wang H. Secular trends and sociodemographic determinants of thinness, overweight and obesity among Chinese children and adolescents aged 7-18 years from 2010 to 2018. Front Public Health. (2023) 11:1128552. doi: 10.3389/fpubh.2023.1128552, PMID: 37213615 PMC10192611

[28] Cohen J., Statistical power analysis for the behavioral sciences. New York, NY: Routledge. (2013) 410–414.

[29] Nakagawa S Johnson PC Schielzeth H. The coefficient of determination R^2^ and intra-class correlation coefficient from generalized linear mixed-effects models revisited and expanded. J R Soc Interface. (2017) 14:20170213. doi: 10.1098/rsif.2017.0213, PMID: 28904005 PMC5636267

[30] Austin PC. Balance diagnostics for comparing the distribution of baseline covariates between treatment groups in propensity-score matched samples. Stat Med. (2009) 28:3083–107. doi: 10.1002/sim.3697, PMID: 19757444 PMC3472075

[31] Zhang Z Kim HJ Lonjon G Zhu Y. Balance diagnostics after propensity score matching. Ann Transl Med. (2019) 7:16. doi: 10.21037/atm.2018.12.10, PMID: 30788363 PMC6351359

[32] Varma S Simon R. Bias in error estimation when using cross-validation for model selection. BMC Bioinformatics. (2006) 7:91. doi: 10.1186/1471-2105-7-91, PMID: 16504092 PMC1397873

[33] Parvandeh S Yeh H-W Paulus MP McKinney BA. Consensus features nested cross-validation. Bioinformatics. (2020) 36:3093–8. doi: 10.1093/bioinformatics/btaa046, PMID: 31985777 PMC7776094

[34] Breusch TS Pagan AR. A simple test for heteroscedasticity and random coefficient variation. Econometrica. (1979) 47:1287–94. doi: 10.2307/1911963

[35] Carroll RJ Ruppert D. Transformation and weighting in regression. New York: Chapman and Hall (1988).

[36] Tellinghuisen J. Weighted least squares in calibration: the problem with using “quality coefficients” to select weighting formulas. J Chromatogr B. (2008) 872:162–6. doi: 10.1016/j.jchromb.2008.07.043, PMID: 18706869

[37] Holmen H Flølo TN Tørris C Torbjørnsen A Almendingen K Riiser K. The role of health literacy in intervention studies targeting children living with overweight or obesity and their parents—a systematic mixed methods review. Front Pediatr. (2025) 12:1507379. doi: 10.3389/fped.2024.1507379, PMID: 39911768 PMC11794496

[38] Ballarin G Galle F Dinacci L Liberti F Cunti A Valerio G. Self-perception profile, body image perception and satisfaction in relation to body mass index: an investigation in a sample of adolescents from the Campania region, Italy. Children. (2024) 11:805. doi: 10.3390/children11070805, PMID: 39062254 PMC11275176

[39] Harrison RN Gaughran F Murray RM Lee SH Cano JP Dempster D . Development of multivariable models to predict change in body mass index within a clinical trial population of psychotic individuals. Sci Rep. (2017) 7:14738. doi: 10.1038/s41598-017-15137-7, PMID: 29116126 PMC5677086

[40] Cheng ER Cengiz AY Miled ZB. Predicting body mass index in early childhood using data from the first 1000 days. Sci Rep. (2023) 13:8781. doi: 10.1038/s41598-023-35935-6, PMID: 37258628 PMC10232444

[41] Delnevo G Mancini G Roccetti M Salomoni P Trombini E Andrei F. The prediction of body mass index from negative affectivity through machine learning: a confirmatory study. Sensors. (2021) 21:2361. doi: 10.3390/s21072361, PMID: 33805257 PMC8037317

[42] Wang Y Zhang L Jiang Y Cheng X He W Yu H . Multiparametric magnetic resonance imaging (MRI)-based radiomics model explained by the Shapley additive exPlanations (SHAP) method for predicting complete response to neoadjuvant chemoradiotherapy in locally advanced rectal cancer: a multicenter retrospective study. Quant Imaging Med Surg. (2024) 14:4617–34. doi: 10.21037/qims-24-7, PMID: 39022292 PMC11250347

[43] Xu J Chen T Fang X Xia L Pan X. Prediction model of pressure injury occurrence in diabetic patients during ICU hospitalization——XGBoost machine learning model can be interpreted based on SHAP. Intensive Crit Care Nurs. (2024) 83:103715. doi: 10.1016/j.iccn.2024.103715, PMID: 38701634

[44] Wang W Zhao Y Li Y. Ensemble machine learning for predicting the homogenized elastic properties of unidirectional composites: a SHAP-based interpretability analysis. Acta Mech Sinica. (2024) 40:423301. doi: 10.1007/s10409-023-23301-x

[45] Tang L Tang C Fu Q Ma C. Predicting travel mode choice with a robust neural network and Shapley additive explanations analysis. IET Intell Transp Syst. (2024) 18:1339–54. doi: 10.1049/itr2.12514

[46] Nguyen HV Byeon H. A hybrid self-supervised model predicting life satisfaction in South Korea. Front Public Health. (2024) 12:1445864. doi: 10.3389/fpubh.2024.1445864, PMID: 39484355 PMC11524807

[47] Wang C Wang Q Ben W Qiao M Ma B Bai Y . Machine learning predicts the growth of cyanobacterial genera in river systems and reveals their different environmental responses. Sci Total Environ. (2024) 946:174383. doi: 10.1016/j.scitotenv.2024.174383, PMID: 38960197

[48] Ramyaa R Hosseini O Krishnan GP Krishnan S. Phenotyping women based on dietary macronutrients, physical activity, and body weight using machine learning tools. Nutrients. (2019) 11:1681. doi: 10.3390/nu11071681, PMID: 31336626 PMC6682952

[49] Kim C Costello FJ Lee KC Li Y Li C. Predicting factors affecting adolescent obesity using general Bayesian network and what-if analysis. Int J Environ Res Public Health. (2019) 16:4684. doi: 10.3390/ijerph16234684, PMID: 31775234 PMC6926973

